# Breast cancer metastasis to the stomach confirmed using gastroscopy: A case report

**DOI:** 10.3892/ol.2014.2260

**Published:** 2014-06-17

**Authors:** LINSHEN TAN, YING PIAO, ZHAOZHE LIU, TAO HAN, FULIN SONG, FEI GAO, YALING HAN, XIAODONG XIE

**Affiliations:** 1 Department of Oncology, Cancer Center of General Hospital of Shenyang Military Area Command, Shenyang, Liaoning 110840, P.R. China; 2 Liaoning University of Traditional Chinese Medicine, Shenyang, Liaoning 110033, P.R. China; 3 Department of Pathology, General Hospital of Shenyang Military Region, Shenyang, Liaoning 110840, P.R. China; 4 Endoscopy Center, General Hospital of Shenyang Military Region, Shenyang, Liaoning 110840, P.R. China; 5 Department of Cardiology, General Hospital of Shenyang Military Region, Shenyang, Liaoning 110840, P.R. China

**Keywords:** breast cancer, infiltrative lobular carcinoma, gastroscopes, metastatic gastric tumor, immunohistochemistry, chemotherapy

## Abstract

Breast cancer metastasis to the stomach is relatively rare. Unlike infiltrating ductal carcinoma, invasive lobular carcinoma (ILC) has a high tendency to metastasize to the stomach. The present study reports a case of a 53-year-old female who had undergone a modified radical mastectomy of the left breast for ILC eight years previously and presented at the clinic seeking treatment for epigastric discomfort from sour regurgitation and belching that had persisted for one month. Gastroscopy revealed multiple apophysis lesions in the stomach, which were diagnosed as metastatic tumors to the stomach. The diagnosis was further established using histological and immunohistochemical analyses for gross cystic disease fluid protein-15, cytokeratin (CK) 7 and CK20. The patient was treated with systemic chemotherapy without surgery. During the treatment, two gastroscopy procedures revealed that the apophysis lesions in the gastric body had narrowed significantly. Few cases of breast cancer metastasizing to the stomach have been reported, particularly those that have been confirmed using gastroscopy. The present study reports a case of breast cancer metastasis to the stomach to raise awareness of the condition.

## Introduction

The most frequent sites of breast cancer metastasis are the local and distant lymph nodes, brain, lung, liver and bone, with metastasis to the stomach being relatively rare. The reported incidence of gastric metastasis in breast cancer patients varies between 2.8 and 27% (1,2). The most common sites of gastric metastasis in breast cancer are the fundus (43%), antrum (43%) or both (14%). In addition, the majority of gastric metastasis are positive for hormonal receptors (79%) (3). Among the various types of breast cancer, those with a lobular pathology have a higher tendency to metastasize to the stomach (4). Differentiating between primary gastric cancer and breast cancer metastasis to the stomach is important for the planning of treatment. The diagnosis is established by histological, gastroscopy and immunohistochemical analysis, but it may be difficult to differentiate primary gastric cancer from gastric metastasis in breast cancer patients. The present study describes a case of breast cancer metastasis to the stomach that was detected using gastroscopy and immunohistochemical analysis. During the treatment, the multiple apophysis lesions in the gastric body were observed to significantly narrow and the symptoms of abdominal discomfort were also alleviated. Patient provided written informed consent.

## Case report

### Patient presentation

A 53-year-old female presented to the Department of General Surgeryof the General Hospital of Shenyang Military Area Command (Shenyang, China) seeking treatment for epigastric discomfort from sour regurgitation and belching that had persisted for one month. The patient had no history of bleeding, tarry stools or associated hemorrhage symptoms. The patient had previously undergone a modified radical mastectomy for an invasive lobular carcinoma (ILC) of the left breast in September 2004. Immunohistochemistry (IHC) revealed that three axillary lymph nodes were involved and that the tissue samples were positive for estrogen receptors (ERs) and progesterone receptors (PRs). Six cycles of paclitaxel (240 mg) were administrated followed by the oral administration of tamoxifen (10 mg) twice daily for five years.

### Diagnosis

**Figure 1:**
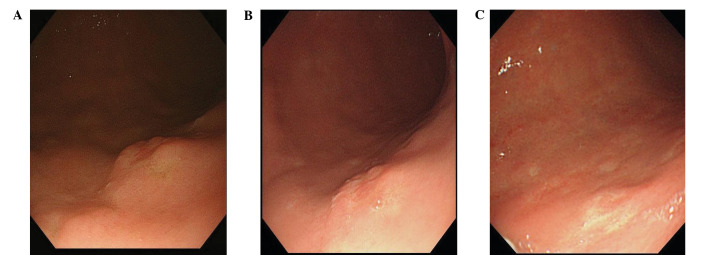
(A) Gastroscopy revealing multiple apophysis lesions in the body of the stomach. (B and C) Gastroscopy following treatment showing that the multiple apophysis lesions were narrowed.

**Figure 2:**
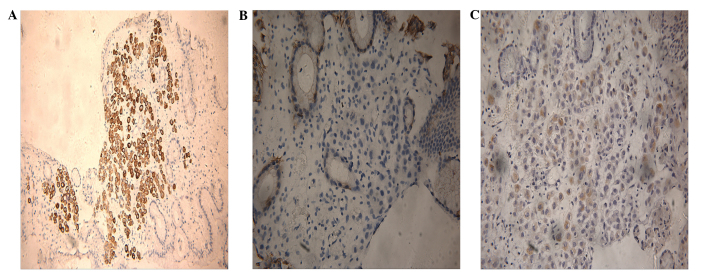
Biopsy samples showing (A) positive immunohistochemistry (IHC) staining for CK7 (stain, H&E; magnification, ×200), (B) negative IHC staininng for CK20 (stain, H&E; magnification, ×400) and (C) positive IHC staining for GCDFP-15 (stain, H&E; magnification, ×400). CK, cytokeratin; GCDFP, gross cystic disease fluid protein.

Gastroscopy revealed multiple apophysis lesions in the body of the stomach (Fig. 1A), which were diagnosed as metastatic tumors of the stomach. Multiple endoscopic biopsies were performed. Few disseminated mild atypia cells were identified in the lamina propria mucosa using hematoxylin and eosin (H&E) staining. Moreover, IHC was performed and showed the tissues to be cytokeratin (CK) 7^+^, CK20^−^ and gross cystic disease fluid protein (GCDFP)-15^+^ (Fig. 2). Based on the medical history of the patient and the findings of IHC and gastroscopy, the patient was diagnosed with breast cancer metastasis to the stomach.

### Treatment

Six cycles of rescue chemotherapy with docetaxel (120 mg) combined with capecitabine (1.5 g orally twice a day) were administered. During the treatment, two gastroscopy procedures revealed that the multiple apophysis lesions in the gastric body were significantly narrowed (Fig. 1B and C). The symptoms of abdominal discomfort were also alleviated. Single drug chemotherapy using capecitabine (1.5 g orally twice a day) was then administered. The patient is currently undergoing follow-up treatment, while continuing capecitabine chemotherapy.

## Discussion

ILC was first described in 1941 by Foote and Stewart (5). ILC accounts for 6–14% of all breast cancer cases and has a distinctive biological behavior (4–7). ILC derives from breast acinar epithelial cells and is the second most common type of breast cancer. Compared with infiltrating ductal carcinoma (IDC), ILC is most likely to occur in older, postmenopausal females with large, well-differentiated, ER-positive tumors and less vessel invasion (5,6,8). Pestalozzi *et al* (6) reported that patients with ILC exhibited a significant early advantage with respect to disease-free survival and overall survival, and that patients with IDC exhibited a significant late advantage after 6 and 10 years, respectively. It was found that the most common sites of breast cancer metastasis were the local and distant lymph nodes, brain, lung, liver and bone, while metastasis to the stomach was relatively rare (6). Previous studies have reported that, unlike IDC, ILC has a higher tendency to metastasize to the stomach, ovaries, meninges, pleura, skin, peritoneum, duodenum and colon (1,3,9,10). This may be associated with the presence of discohesive small cells, a phenotypic trait that characterizes ILC. The loss of E-cadherin, which is observed in the majority of ILC cases, may lead to changes in cell-cell adhesion and preferential growth at sites of metastasis (8).

The metastasis of primary breast cancer to the stomach is particularly uncommon at the time of the initial diagnosis (3,11). Symptoms of gastric metastasis are often non-specific and include epigastric pain, anorexia, non-fatal hemorrhage, vomiting and dysphagia (2,12). Substantial variability is shown by the endoscopic findings, including the observation of lesions with a benign appearance or primary gastric cancer (3,10,13) with diffusely infiltrative lesions (2). Gastric metastases are usually recognized as a diffuse infiltration by endoscopy (2). Endoscopic biopsies histologically confirm ~90% of gastric metastatic lesions (12). Immunohistochemical analyses are recommended for the accurate diagnosis of breast cancer metastasis to the stomach. ER and PR expression are not observed in gastric cancer and are useful for diagnosing breast cancer metastases to the stomach (14). However, if the primary lesion is negative for ER and PR, these markers are not useful for diagnosing breast cancer metastases in the stomach (15). Immunostaining of CK20 and CK7 also aids in the formation of a diagnosis (15). The immunoperoxidase technique has revealed that the negative expression of GCDFP-15 is found in benign and malignant lesions of the stomach. Thus GCDFP-15 has been proposed to be a specific tissue marker of apocrine epithelium and breast carcinomas with apocrine features (16). Although certain methods are used to diagnose gastric metastases, the diagnosis is difficult. The identification of gastric metastases using endoscopy is also hard. Furthermore, false-negative IHC results are common, as the tumor cells are scattered and located in deep mucosal tissue following pathological biopsy (2,3,10,12,13). In the present study, the endoscopic findings of the patient showed typical metastases with diffuse intramural infiltration to the stomach.

The treatment recommendation for gastric metastases from breast cancer is typically a systemic treatment. Surgical intervention should be reserved for palliation or certain cases of solitary resectable gastrointestinal tract metastases (17).

The patient discussed in the present study was treated with docetaxel combined with capecitabine, and following six cycles of rescue chemotherapy, gastroscopy revealed that this treatment had induced a marked result. The patient also experienced partial remission of the abdominal discomfort and an overall improvement in the quality of life.

The present case represents a typical metastatic tumor to the stomach, which was detected using gastroscopy eight years after the surgical removal of ILC. It is essential to use GCDFP-15 and CK7/20 immunostaining of the biopsy tissue in order to identify breast cancer metastases in the stomach.

When a patient has a history of ILC, endoscopic examinations should be performed carefully. Moreover, physicians should provide the clinical history of the patient to the endoscopist and the endoscopist should provide sufficient information to the pathologist in order to obtain an accurate diagnosis of breast cancer metastasis to the stomach and improve the patient’s quality of life.
